# Continuous Auricular Electroacupuncture Can Significantly Improve Heart Rate Variability and Clinical Scores in Patients with Depression: First Results from a Transcontinental Study

**DOI:** 10.1155/2013/894096

**Published:** 2013-11-12

**Authors:** Xian Shi, Gerhard Litscher, Huan Wang, Lu Wang, Zengkai Zhao, Daniela Litscher, Jingqiao Tao, Ingrid Gaischek, Zemin Sheng

**Affiliations:** ^1^Department of Acupuncture, People's Liberation Army General Hospital, Beijing 100853, China; ^2^Stronach Research Unit for Complementary and Integrative Laser Medicine, Research Unit of Biomedical Engineering in Anesthesia and Intensive Care Medicine, and TCM Research Center Graz, Medical University of Graz, 8036 Graz, Austria; ^3^Privatclinic Lassnitzhöhe, 8301 Lassnitzhöhe, Austria

## Abstract

The goal of this study was to investigate the impact and acceptability of providing continuous auricular electroacupuncture as an adjunct to conventional medications for patients with depression. Ten patients with a mean age ± SD of 43.3 ± 10.4 years were able to provide informed consent. The quantitative and qualitative outcome measures were heart rate, heart rate variability (HRV), and different clinical scores. The study documented that a special kind of auricular electro acupuncture, applied over a period of three days, can improve various aspects of quality of life significantly but also highlighted the significant increase of HRV whilst having acupuncture treatment. In conclusion, our study shows stimulation-related and quantifiable clinical and physiological alterations in parameters after continuous auricular acupoint stimulation in patients with depression. Improved access to electro acupuncture treatment would be of major benefit for these patients. Further studies are necessary in order to verify the gained results.

## 1. Introduction

A review article from 2011 summarizes 30 randomized controlled trials which evaluated manual acupuncture, electroacupuncture, or laser acupuncture in nearly 3000 patients with major depressive disorder [1]. No consistent benefit was noted with any form of acupuncture [1]. However, our research group recently found acute stimulation effects on neurovegetative parameters like heart rate (HR) and heart rate variability (HRV) in patients with depression [2, 3] and insomnia [4].

In previous studies it has also been shown that both the autonomic and the central nervous system could be modified by auricular vagal stimulation via projections from the auricular branch of the vagus nerve to the nucleus of the solitary tract [5]. Auricular acupuncture is proposed to prevent neurodegenerative diseases via vagal regulation [5]. However, there is a controversy on the specificity and the efficacy of auricular acupoints for treating diseases [5].

An innovative concept of the current teleacupuncture technology has been implemented at the Traditional Chinese Medicine (TCM) Research Center Graz in Austria (http://litscher.info/ and http://tcm-graz.at/) in 2010 in cooperation with different institutions in China over a distance of several thousands of kilometres [2, 6–8]. The present clinical study was performed at the Military Acupuncture Centre at the People's Liberation Army General Hospital in Beijing, in cooperation with the Austrian center mentioned above.

This research describes results from continuous auricular electroacupuncture measurements in patients with depression using computer-based HRV recordings before, during, and after long-lasting electroacupuncture under standardized clinical conditions in Beijing, China. All data analyses were performed in Graz, Austria.

## 2. Subjects and Methods

### 2.1. Patients

Ten patients (7 females, 3 males; mean age ± SD 43.3 ± 10.4 years; range 29–59 years) suffering from depression (Chinese diagnosis “Yu Zheng”) and therefore receiving acupuncture treatment were investigated at the Chinese People's Liberation Army Hospital in Beijing. The experiment started in all patients at the morning. The clinical evaluation of the patients, performed immediately before the first HRV data recording, used three main scales: the Hamilton anxiety rating scale (HAM-A) [9], the Athens insomnia scale (AIS) [10], and the Hamilton rating scale for depression (HRSD) [11]. No patient was under the influence of centrally active medication and had a history of heart or cerebrovascular disease, respiratory or neurological problems, or hypertension. The study was approved by the ethic committee of the Chinese People's Liberation Army Hospital and carried out in compliance with the Declaration of Helsinki. All patients with depression gave oral informed consent.

### 2.2. Biosignal Recording in China and Data Analysis in Europe

The duration of RR-intervals is measured during a special time period (5 min), and on spectral analysis basis HRV is determined. Electrocardiographic (ECG) registration is performed using three adhesive electrodes (Skintact Premier F-55; Leonhard Lang GmbH, Innsbruck, Austria) which are applied to the chest.

For the joint investigations, the researchers in China used a medilog AR12 HRV (Huntleigh Healthcare, Cardiff, United Kingdom) system from the TCM Research Center at the Medical University in Graz. The system has a sampling rate of 4096 Hz [12], the raw data are stored digitally on a CompactFlash memory card, and after removing the card from the portable system, the data were read by a card reader connected with a standard computer in China and then transferred to the TCM Research Center Graz via internet. The biosignals were analyzed and HRV was displayed in a way to help to judge the function of the autonomic nervous system [6–8].

Similar to previous studies [2–4, 12–14], mean HR, total HRV, and the LF (low frequency)/HF (high frequency) ratio of HRV were chosen as evaluation parameters, as such being recommended by the Task Force of the European Society of Cardiology and the North American Society of Pacing and Electrophysiology [15].

### 2.3. Auricular Electroacupuncture (Punctual Stimulation) and Procedure

A method for ear acupuncture is the electrical point stimulation system (P-Stim; Biegler, Austria). Ultrathin permanent needles are applied at the ear. A generator, located behind the acupunctured ear, produces electrical stimulation impulses (using a constant AC current of 1 mA; impulse duration: 1 ms; stimulus frequency: 1 Hz; alternating 15 min active stimulation and 15 min break (no active stimulation) over a period of three days), which are transferred to the acupuncture areas via the needles. The ear is chosen because a concentration of free nerve ends/acupuncture points are located here [16]. P-Stim allows continuous, intermitting stimulation up to several days combined with absolute mobility of the patient.

Selected acupuncture points at the ear were chosen by an experienced Chinese acupuncturist. A position tape previously prepared with the P-Stim application pointer was applied. This procedure was repeated until all acupuncture points were marked. Then, the needles could be taken up by the application pointer and applied.

A Chinese physician adhered the actual device behind the acupunctured ear with the integrated adhesive electrode. Then, the wires were connected to the permanent needles by snapping over the plastic rings, and everything was fastened with adhesive tape. Finally, P-Stim was activated by removing the adhesive foils from the batteries and by opening the lid. Further methodological details are described in previous publications [17–19].

**Figure 1:**
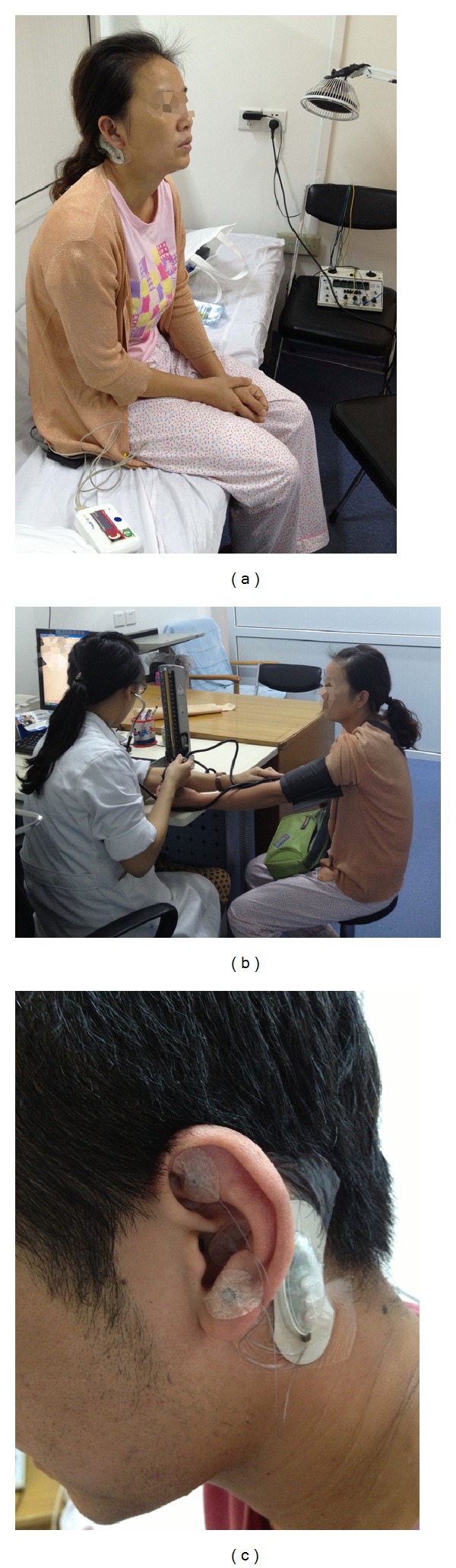
Ear acupuncture using P-Stim in Beijing (a)–(c).

The following ear acupoints were used in this study: Shenmen, Small Intestine, and Heart (see also Figure 1(c)).

**Figure 2:**
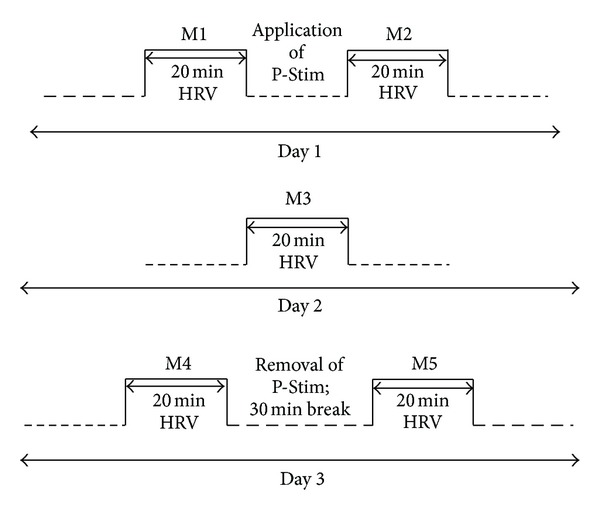
Measurement procedure.

Patients had to come to the clinic on three consecutive days. On day 1, they first answered the score questionnaires; then a first HRV measurement (M1 in Figure 2) was performed for 20 min. After that, the P-Stim auricular stimulation equipment was applied and activated, and immediately after that, a second HRV measurement (M2 in Figure 2) took place. On the second day, the patient came to the clinic again, still wearing the P-Stim device; the questionnaire was answered again and a third HRV measurement (M3 in Figure 2) was recorded. And finally, on day 3, the patient came again, still wearing the auricular punctual stimulation. A fourth HRV measurement (M4 in Figure 2) was performed before the P-Stim device was removed. After a break of 30 min, a last HRV measurement (M5 in Figure 2) followed, and the questionnaire was answered one last time.

### 2.4. Statistical Analysis

Data were analyzed using SigmaPlot 11.0 software (Systat Software Inc., Chicago, USA). Graphical presentation of results uses box plot illustrations. Testing was performed with Friedman repeated measures ANOVA on ranks and Tukey or Holm-Sidak test. The criterion for significance was *P* < 0.05.

## 3. Results

**Figure 3:**
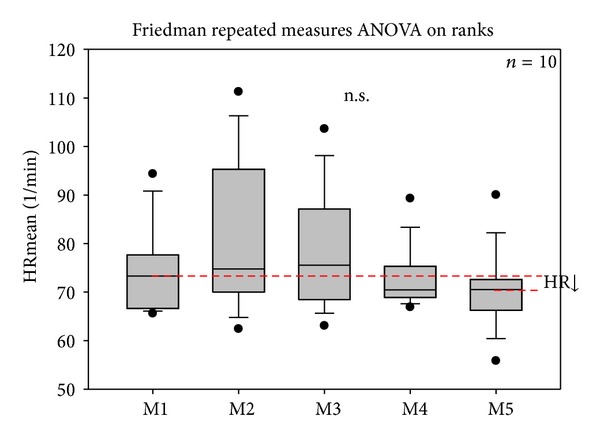
Box plots displaying the changes in mean heart rate (HR) of the 10 patients. After the fifth measurement, on the third day, HR had decreased; however, the changes were not significant. The ends of the boxes define the 25th and 75th percentiles with a line at the median and error bars defining the 10th and 90th percentiles.

**Figure 4:**
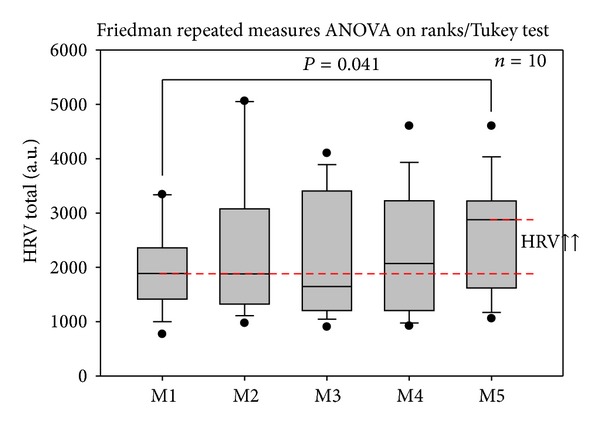
Changes in total heart rate variability (HRV). Electrical auricular stimulation induced a significant increase in total HRV in the ten patients investigated in this study. For further explanations, see Figure 3.

Figures 3 and 4 show the mean HR and total HRV from the ECG recordings of 10 patients with depression during the five measurement phases (M1–M5). There was a slight decrease in HR, but no significant change before, during, or after the stimulation sessions.

In contrast to HR, HRV increased significantly (*P* = 0.041) after continuous auricular electroacupuncture over a period of three days (see Figure 4, M5).

**Figure 5:**
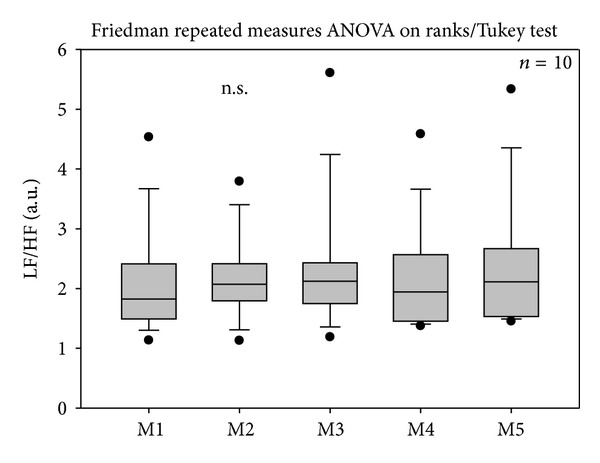
The low frequency (LF)/high frequency (HF) ratio did not change significantly during the three days of the investigation. For further explanations, see Figure 3.

Furthermore, continuous HRV monitoring showed no significant alterations in the LF/HF ratio during or after acupuncture stimulation at the ear (see Figure 5).

**Figure 6:**
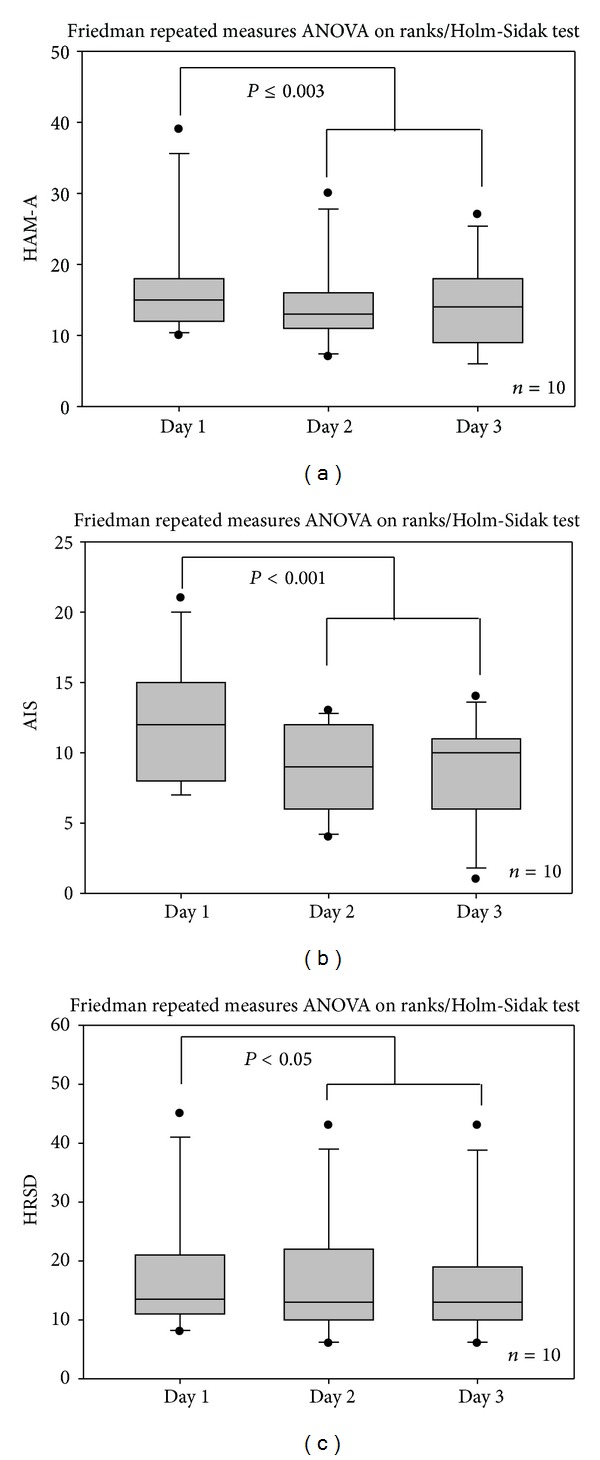
Significant decreases of the three scores investigated within this study. (a) Hamilton anxiety rating scale (HAM-A); (b) Athens insomnia scale (AIS); (c) Hamilton rating scale for depression (HRSD). For further explanations, see Figure 3.

The analysis of the three clinical scores revealed interesting results. In all scores there was a significant reduction already after the first day of continuous electroacupuncture with P-Stim (see Figures 6(a)–6(c)).

The data of the blood pressure values of all 10 patients showed insignificant results (systolic blood pressure (mean ± SD): M1: 107.5 ± 10.1 mmHg, M5: 105.6 ± 9.9 mmHg; diastolic blood pressure (mean ± SD): M1: 73.8 ± 7.4 mmHg, M5: 72.1 ± 5.5 mmHg).

## 4. Discussion

Depression is one of the most disabling diseases in Europe and Asia, causing a significant burden both to the individual and to society [20, 21]. The World Health Organization (WHO) data suggests that depression causes 6% of the burden of all diseases in Europe. Already in 2006, at least 21 million people were affected by depression in 28 European countries (with an overall population of 466 million) [20]. The total annual cost of depression in Europe in 2004 was estimated to be 118 billion Euro [20], which corresponds to costs of 253 Euro per inhabitant [20].

A recent survey in China [21] indicated that the 12-month prevalence rate of depressive disorders was 2.5% in Beijing and 1.7% in Shanghai. These disorders may result in disability, premature death, and severe suffering of those affected and their families. The total estimated cost of depression in China is about 6,264 million US dollars (USD, at 2002 prices). Direct costs were 986 million USD, which corresponds to about 16% of the total cost of depression. Indirect costs were, accordingly, 5,278 million USD, or 84% of the total cost of depression [21].

One important way to stop this cost explosion in Europe and China is through increased research efforts in this field. Better detection, prevention, treatment, and patient management are imperative to reduce the burden of depression and its cost [20].

In order to achieve better outcome for the patients, it is mandatory that scientists develop new strategies and that physicians have an up-to-date knowledge of recent advances in evidence-based complementary medicine.

Auricular acupuncture is a special kind of stimulation which is slowly becoming accepted as an evidence-based complementary medical treatment method [16, 17]. Electroacupuncture at auricular points is also used in patients with depression [22, 23]. Especially continuous electrical stimulation of acupoints over several days can increase the effects of acupuncture [17–21, 24–29].

This study introduces first measurements in patients with depression using a miniaturised system for continuous electric acupoint stimulation at the ear. In previous studies, the influence on cerebral functions could be observed [17, 28]. One of the main advantages of the P-Stim system is the complete mobility for the patients, which is not the case when using other systems. In addition to treating patients with neurological diseases like depression, this concept seems to be useful for treating addiction, allergies and in special areas of anaesthesiology [24–29].

Results of a clinical point stimulation study show that a marked decrease in VAS (Visual Analogue Score) of pain occurred in 31 persons who underwent electric stimulation [17, 30]. The average stimulation duration was 36.6 hours (treatment time range: 18–72 hours). A significant decrease in VAS during stimulation was observed in 67% of the patients, and 29% of the patients reported a moderate reduction in pain. A relevant reduction in pain medication was observed in 70% of the patients. No accompanying medication was necessary in half of the patients treated. Almost all patients reported an improvement in their general health situation [17, 30]. This is in accordance with the results of our present study. All clinical scores (HAM-A, AIS, HRSD) showed a significant improvement already after 24 hours of continuous electrical stimulation. In addition, HRV, which is a reliable indicator of the state of health [2–4, 6, 7], also improved significantly.

In conclusion, our study shows stimulation-related and quantifiable clinical and physiological alterations in parameters after continuous auricular acupoint stimulation in patients with depression. Further studies are necessary in order to verify the gained results.
